# Effectiveness of Ergonomic Training to Decrease Awkward Postures during Dental Scaling Procedures: A Randomized Clinical Trial

**DOI:** 10.3390/ijerph182111217

**Published:** 2021-10-26

**Authors:** Ana Virginia de Santana Sampaio Castilho, Edgard Michel Crosato, Silvia Helena de Carvalho Sales-Peres, Gerson Aparecido Foratori Junior, Adriana Rodrigues de Freitas Aznar, Rogerio Leone Buchaim, Daniela Vieira Buchaim, Dayane Maria Braz Nogueira, Eliana de Souza Bastos Mazuqueli Pereira, Luis Carlos Paschoarelli, Eliel Soares Orenha

**Affiliations:** 1 Department of Pediatric Dentistry, Orthodontics and Public Health, Bauru School of Dentistry (FOB/USP), University of São Paulo, Bauru 17012-901, SP, Brazil; anavcastilho@usp.br (A.V.d.S.S.C.); shcperes@usp.br (S.H.d.C.S.-P.); gerson.foratori@usp.br (G.A.F.J.); adrianafreitas@usp.br (A.R.d.F.A.); 2 Social Dentistry Department, School of Dentistry (FOUSP), University of São Paulo, São Paulo 05508-000, SP, Brazil; michelcrosato@usp.br; 3 Dentistry Course, Faculty of the Center West of São Paulo (FACOP), Piratininga 17499-010, SP, Brazil; 4 Department of Biological Sciences, Bauru School of Dentistry (FOB/USP), University of São Paulo, Bauru 17012-901, SP, Brazil; rogerio@fob.usp.br; 5 Graduate Program in Anatomy of Domestic and Wild Animals, Faculty of Veterinary Medicine and Animal Science, University of São Paulo (FMVZ/USP), São Paulo 05508-270, SP, Brazil; 6 Postgraduate Program in Structural and Functional Interactions in Rehabilitation, Postgraduate Department, University of Marilia (UNIMAR), Marília 17525-902, SP, Brazil; danibuchaim@alumni.usp.br (D.V.B.); elianabastos@unimar.br (E.d.S.B.M.P.); 7 Teaching and Research Coordination of the Medical School, Medical School, University Center of Adamantina (UniFAI), Adamantina 17800-000, SP, Brazil; 8 Department of Prosthodontics and Periodontics, Bauru School of Dentistry (FOB/USP), University of São Paulo, Bauru 17012901, SP, Brazil; dayanenogueira@usp.br; 9 Department of Design, School of Architecture, Arts, Communication and Design, São Paulo State University (UNESP), Bauru 17033-360, SP, Brazil; luis.paschoarelli@unesp.br

**Keywords:** dental ergonomics, training, awkward postures, dental scaling

## Abstract

Studies demonstrate that there is a lack of effective ergonomic principles for adopting a neutral posture during the execution of dental procedures. ISO 11.226:2000 Standard, Corr. 1:2006 has been thoroughly evaluated and adapted to the way that dentists work by the European Society of Dental Ergonomics (ESDE). However, after 15 years, no studies that showed strong evidence of effectiveness in reducing the prevalence of awkward posture in applying its parameters within the scope of dental practice were found. The aim of this study was to verify the effectiveness of applying the ergonomic parameters proposed by the European Society of Dental Ergonomics (ESDE) and ISO 11226 in reducing the prevalence of the main awkward postures adopted by female dental surgeons during the execution of dental scaling on a dental mannequin. A randomized clinical trial was carried out with sixty dental surgeons randomly assigned to two groups: the intervention group, who received instructions and theoretical and practical ergonomic training; and the control group, who received the same training only at the end of the study. For data analysis, Software IBM SPSS 27 and RStudio was used. Descriptive statistics were performed to verify the effectiveness of the intervention, and generalized linear models (specifically, generalized estimated equation models) were used. Poisson distribution was carried out with log link function and network analyses. Sixty female dental surgeons participated in the study. Twenty-two were distributed in the intervention group and thirty-eight in the control group. It was found that ergonomic training enabled a 63% reduction in the prevalence of awkward postures and that there was a statistically significant difference (*p* < 0.001) only in the intervention group. The analyses showed that the estimated marginal means of postures not recommended in the groups’ initial control, final control, initial intervention, and final intervention were 8.6, 8.2, 9.0, and 3.4, respectively. The relationship of networks analyses of the variables is shown with different profiles in the control and intervention groups, but the same pattern between the groups only vary in the strength and direction of the correlations. It was concluded that the ergonomic training based on the parameters of ISO 11226 and DIN EN 1005-4, and its adaptations to the dental practice provided by the European Society of Dental Ergonomics, as well as recent studies, contributed significantly to reducing the prevalence of awkward postures adopted by female dentists during the simulation of the basic periodontal procedures; however, it was not effective enough to improve the posture of the head and neck.

## 1. Introduction

Knowledge about ergonomics has advanced a lot. Studies have revealed its importance for the quality of life of the dentist. However, there is a great lack of application of its concepts and principles in dental practice [1,2,3,4,5,6]. Scientific evidence indicates the high prevalence of joint, muscle, lumbar problems, and other work-related musculoskeletal disorders (WMSD), mainly due to poor posture, lack of ergonomic planning of equipment, work environment, work systems, among others [2,7,8,9,10,11,12,13]. This has caused many dentists to work with low productivity, low comfort, and mainly without quality of life, which, in many cases, may temporarily leave them unemployed or even condemn them to abandon their career early [8,9].

Among the main causes of the development of WMSD in dentistry are poor posture at work. A study carried out with 1250 dentists from Belgium, Luxembourg, and the Netherlands showed that 64% of the evaluated professionals had disorders in the neck, shoulders, and spine; 42% suffered from headaches; there was a higher occurrence of posture disorders in women; greater occurrence of disorders in dentists over 1.80 m tall; greater occurrence of neck and shoulder disorders among dentists who worked with direct view of the maxilla; and 1/3 of dentists had muscle fatigue. It was also demonstrated that the more difficult the treatment is, the worse the posture, and also that the neck muscles were the ones that most contributed to the occurrence of fatigue and disorders [13]. Several studies showed that dentists worked in the same posture for many hours at a time and were very exposed to static body posture. In addition, they use equipment with inadequate lighting and color combinations and are exposed to an irritating sound load, which affects both mental and physical health [2,4,9,11,13].

Jianru et al. found that there was a high prevalence of WMSDs in the neck, trunk, and lumbar region. They also found that professionals who worked in the specialty of periodontics had the biggest complaints in most parts of the body, with the exception of the wrist and knees. The authors concluded that WMSD prevention should be specific to the characteristics of each type of specialty and that risk factor prevention should be introduced as early as possible [14].

Currently, there are two theories that guide the adoption of the dentist’s working posture in the international scientific literature. The first of them, called classical theory, has been widely used for over half a century, which is defended and predominantly applied in ergonomic training in dentistry courses, both in Brazil and in several other countries around the world [5,15,16]. This theory proposes a working posture so that the dentist’s legs maintain a 90-degree angle in the popliteal region and between the thighs and the abdomen. Furthermore, for the right-handed dentist, this theory proposes that only the dentist’s left leg be positioned under the backrest of the patient dental chair and that his right leg remains parallel to the patient dental chair, while the opposite is indicated for the left-handed dentist. Unlike the classical theory, the second theory, more recently proposed by ESDE based on the parameters described in ISO 11226, proposes a seated working posture in which there is a minimum angle of 110 degrees in the popliteal region and between the thighs and abdomen of the dentist. In addition, it also proposes that the dentist’s two legs be under the backrest of the patient dental chair, which allows better approximation and freedom of movement for dentists to suit their visual and approach needs in the intra-oral work field. Thus, according to ESDE [17], the dentist is allowed to adopt a more dynamic posture, and with greater freedom of approach and movement of the dentist’s body around the chair. Consequently, the need for excessive flexion of the upper limbs is reduced, facilitating the adoption of a neutral working posture and with less muscle and skeletal overload. When, for example, the dentist performs dental scaling procedures, it is necessary to visualize the mesial, distal, buccal, and lingual/palatal dental surfaces, which are directed in opposite directions. If there is no freedom of dentist’s body movement so that the patient dental surface is positioned in front of the dentist’s eyes, the dentist will automatically lean excessively, adopting an awkward posture.

ISO 11.226:2000 Standard, Corr. 1:2006 has been thoroughly evaluated and adapted to the way the dentist works by the European Society of Dental Ergonomics (ESDE). However, after 15 years, no studies were found that showed strong evidence of effectiveness in reducing the prevalence of awkward posture in applying its parameters within the scope of dental practice. Due to the fact that the classical theory has been applied for a long time and the fact that there is no strong scientific evidence, on most dentistry courses, ergonomic training is still based on classical theory, contrary to the requirements and parameters for ergonomic posture proposed by ESDE and ISO 11226. The change in the theoretical foundation in training to adopt a neutral posture at work by dentists is also hampered by the little scientific evidence of the contribution provided by educational interventions on work postures and the prevalence of WMSD.

According to studies by Lindegard et al., Elders et al., Linton and Van Tulder, and Veiersted et al. [18,19,20,21], there are inconsistencies, and the scientific evidence of the positive effects of educational interventions on work postures and the prevalence of WMSD is not strong. Studies have shown that there is no evidence that educational interventions in the workplace are effective in reducing complaints and improving performance [22,23]. The great concern about the high prevalence of WMSD and the difficulties related to learning and adopting a healthy work posture led the European Society of Dental Ergonomics—ESDE to publish the document: “Ergonomic requirements for dental equipment. Guidelines and recommendations for the design, construction and selection of dental equipment”. These requirements establish the basis for teaching ergonomics at universities, for the design, construction, and selection of dental equipment, and information for dental surgeons to adopt a safe, comfortable, and healthy posture when providing dental care to their patients [17,24].

The close relationship between the disorders that affect dental professionals and their routine activities justifies the relevance of training dentists according to their specialty, to avoid the related symptoms to WMSD and its consequences [14]. It is necessary to make dentists aware of preventive measures that can be developed and planned against these disorders, so that they can enjoy a better quality of life [1,2,3,10,14,15,18]. Systematic reviews of the literature carried out by Mulimani et al. [16] highlight that, in the dental field, there are no studies with strong scientific evidence that ergonomic training is effective for the prevention of work-related musculoskeletal disorders. Similarly, the scientific evidence for the positive effects of ergonomic training on work postures is not strong, and the results of observational studies and rare studies are inconsistent.

Given the above, due to the gap in the area, the aim of this study was to verify the effectiveness of the application of training based on the ergonomic parameters proposed by the European Society of Dental Ergonomics (ESDE) and ISO 11226 in reducing the prevalence of the main awkward postures adopted by female dentists who apply the fundamentals of classical theory during execution of dental scraping on a dental mannequin. Hypotheses of this study are: H0 = Ergonomic training based on ESDE, ISO 11226, and DIN EN 1005-4 parameters which do not interfere with the prevalence of awkward postures; and H1 = Ergonomic training based ESDE, ISO 11226, and DIN EN 1005-4 parameters which reduce the prevalence of awkward postures.

## 2. Materials and Methods

### 2.1. Study Type

A randomized clinical trial was undertaken for this study. For the preparation of this document, the Consort Guidelines were followed [25]. A fulfilled check-list was provided as a supplementary material (Table S1). This clinical trial has protocol number #11919 in the Brazilian Clinical Trials Registry (REBEC) and can be accessed at: https://ensaiosclinicos.gov.br (accessed on 17 October 2021).

### 2.2. Ethical Considerations

This study was evaluated and approved by the Ethics Committee in Research with Human Beings of the Bauru School of Dentistry, University of São Paulo, Bauru (São Paulo, Brazil) (CAE: 38821714.4.0000.5417).

### 2.3. Study Population and Eligibility Criteria

One hundred and eighty (180) female dentists, who were enrolled in training courses at specialization, Master’s, or doctoral level, were invited to participate in the study.

Inclusion criteria were having completed a dental course at least 2 years ago, female, and being enrolled in one of the postgraduate courses in the macro region of the city of Bauru, State of São Paulo, Brazil.

Exclusion criteria were: male dentist, neither presenting nor having presented MSD symptoms during the last seven days, not being in the final stages of the gestation period, and that the expected date for completion of the course only occurred after the end of data collection in the final phase (post training).

### 2.4. Study Sample, Sample Size and random Allocation Procedure

Sixty-eight (68) of the one hundred and eighty (180) invited dentists accepted the invitation to participate in the study (positive response rate of 38%). This study was conducted only with female dentists because they are at increased risk of developing MSD [26]. It was considered unfeasible to obtain a sample number of male dentists in the courses that was sufficiently representative for one more variable to be considered in the study. Hence, the research participants were all female dentists, with completion of the dental course of at least 2 years. The minimum age of participants was 24 years and the maximum were 45 years, with a mean of 27.48 years (SD 4.62 years). All of them were graduates in dentistry and were enrolled in one of the specialization courses, i.e., Masters or doctorates at the Faculty of Dentistry of Bauru (FOB/USP), or in Dental Education Institutes, Training Centres, and Class Associations in the region of Bauru, São Paulo, Brazil.

The random allocation of participants to each group was performed by two researchers (ESO and AVSSC) in an attempt to ensure that the researcher responsible for assessing outcomes and data analysing was blinded. Random allocation procedure was performed by using the Excel software of the Office Package version 2019. First, a code was assigned to each participant, numbering from 1 to 68. Then, in an Excel spreadsheet, a column labelled as “ID_Part” was created, and then a number sequence was created in the lines of that same column between 1 and 68. Then, another column labelled as “Group” was created, and, by applying the function “=RANDBETWEEN(0;1)”, the codes 0 or 1 were assigned to each line. Participants who received code zero were allocated to the control group and those who received code 1 were allocated to the intervention group. Finally, a descending classification was made based on the column group, where it was found that 29 participants were assigned to the intervention group. Therefore, the code of the first participant was manually changed from code 0 to code 1, so that 30 participants were assigned to the intervention group and 38 to the control group, resulting in allocation ratio of 0.8.

The quantitative dependent variable was obtained by the total sum of awkward postures; thus, data analysis was performed using generalized linear models, applying the generalized linear models method, the generalized estimated equation, with Poisson distribution and with log link function. Thus, 20 participants per group were determined as the minimum sample size [27]. Considering the possibility of losses due to participant abandonment during the research period, 30 participants were decidedly allocated to the intervention group and 38 to the control group.

**Figure 1:**
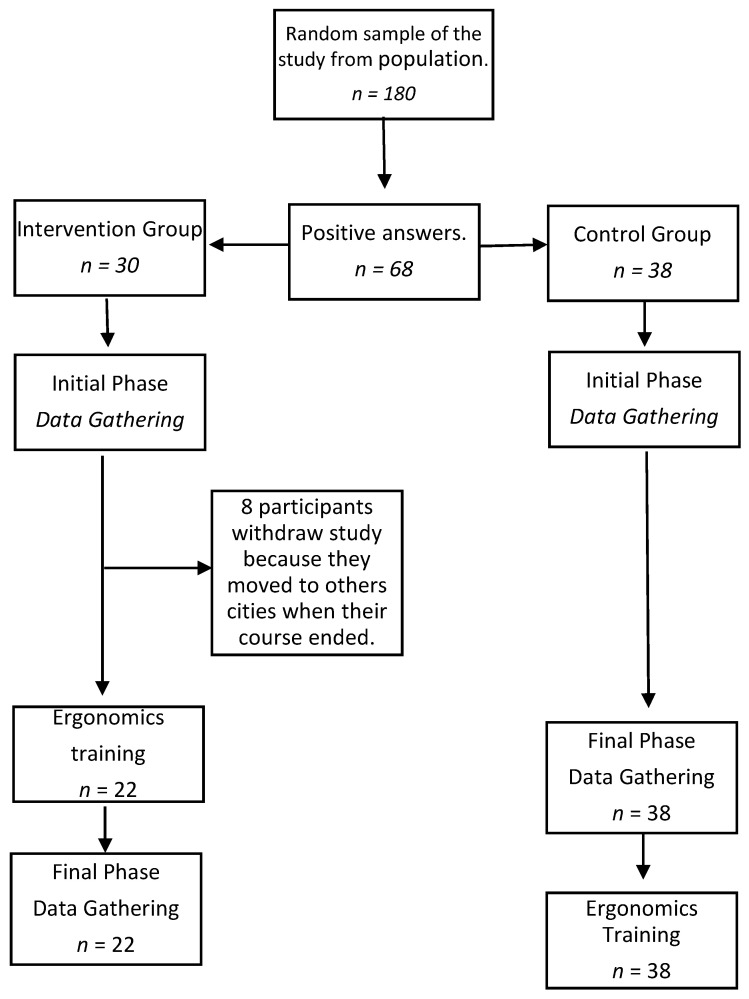
Flow chart of participants enrolled in the study and the logistics regarding the two groups.

Figure 1 presents flow chart of participants enrolled in the study and the logistics regarding the two groups, as follows:

- CG (control)—composed of 38 professionals who received training only at the end of the study.
- IG (intervention)—initially composed of 30 professionals, 8 of whom declined to participate in the research for various reasons, and 22 professionals remained so. These were given the theoretical and practical ergonomic training proposed by ESDE (Supplementary Material). This training was based on the principles described in the document “Ergonomic requirements for dental equipment. Guidelines and recommendations for designing, producing and choosing dental equipment” [17]. This document was based on the Norms ISO 6385: 2004 (E), DIN EN 1005-4, and ISO 11226: 2000-Cor.1: 2006 (E), and their applicability to the dental practice [28,29,30].

### 2.5. Phase 1 (Baseline)

This phase was carried out from March to September 2016.

No ergonomic guidelines were given regarding the professional’s posture or how to correctly position of the work field in this phase.

**Figure 2:**
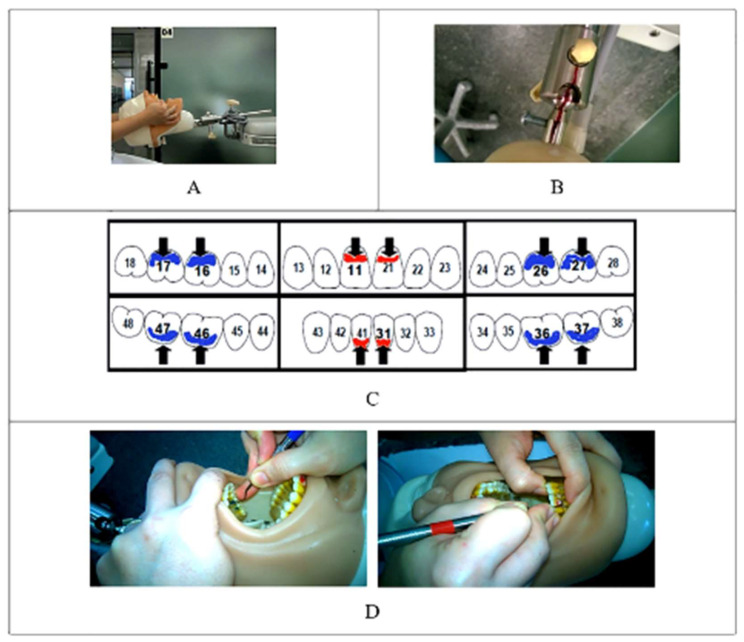
Attachment of the phantom head to the patient’s dental chair and organization of the dental procedure. (**A**) Phantom head attachment to the patient’s dental chair; (**B**) Control of the phantom head motion by means of boundary lines; (**C**) Dental surfaces to be cleaned; (**D**) Identification of anterior and posterior curettes by means of colors.

A phantom head (Pronewodonto^®^—BOB model) was adapted to be properly attached to the patient’s dental chair (Figure 2A) to allow the necessary adjustment of height and head movements in three directions, as proposed by ESDE [17].

The participants were instructed and monitored regarding to the limits of motion of the phantom head (Figure 2B). Participants in both groups were asked to perform dental scaling procedures on six dental surfaces: buccal surfaces of teeth 16, 11, 26, and 31, and lingual surfaces of teeth 36 and 46 (Figure 2C). To facilitate the control and identification of the proper use of each dental curette to each tooth surface, the tooth surfaces were previously covered with red nail enamel for anterior teeth and a blue one for posterior teeth. Dental curettes were also identified with the same colors according to the indications for use on each surface to be cleaned (Figure 2D).

**Figure 3:**
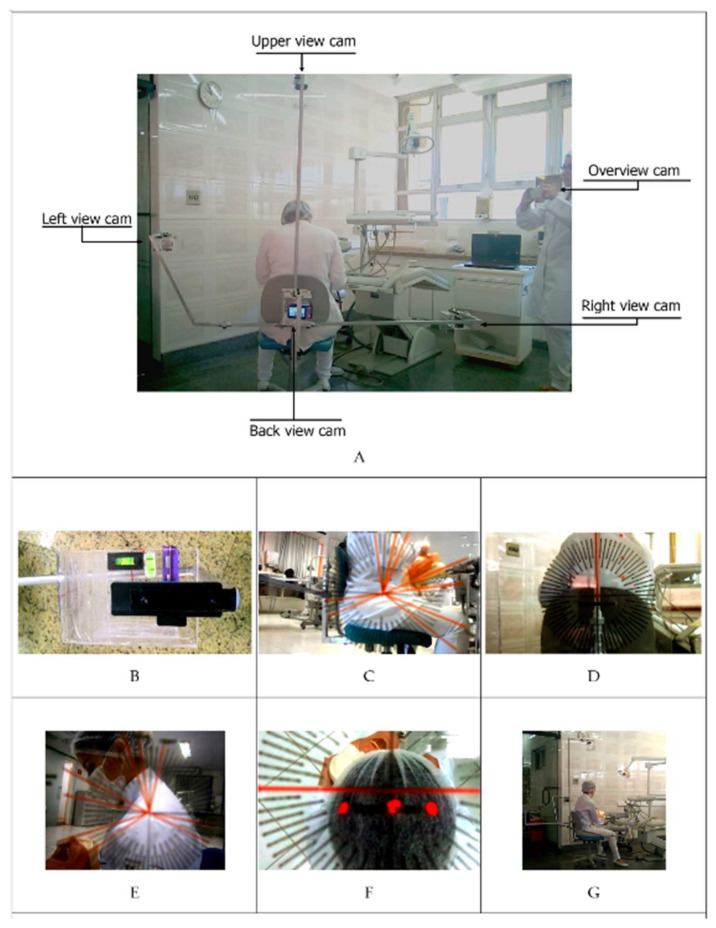
Device built to support cameras 1 to 4, camera 5 positioning and views obtained by each of the cameras. (**A**) Positioning of the 5 cameras; (**B**) Transparent acrylic support with the parameters and limits of angular inclinations and bubble levels to keep cameras straight; (**C**) Right view; (**D**) Back view; (**E**) Left view; (**F**) Upper view; (**G**) Overview camera.

All observations were performed by using 5 video cameras (Figure 3A), fixed 1 m away from the research participant, with the exception of Camera 5, which was moved by the researcher at a certain variable distance. Table 1 shows parameters analyzes according cameras. Camera 1 (right side view—Figure 3C): arching of the trunk or convex lumbar lordosis (parameter 4), the frontal inclination of the trunk (parameter 5), arm flexion (requirement 9), flexion of the forearms (parameter 11), and the angulation of the popliteal region (parameter 12). For Camera 2 (Back view—Figure 3D), the following were evaluated: lateral trunk flexion (parameter 6), shoulders raised (parameter 8), and abduction of the arms (parameter 10). For Camera 3 (left side view—Figure 3E), the following were evaluated: the frontal inclination of the head (parameter 2) and neck flexion (parameter 3). For Camera 4 (upper view—Figure 3F), the following were evaluated: lateral flexion and rotation of the head (parameter 1), and trunk rotation (parameter 7). The leveling of the cameras was carried out and monitored constantly with bubble level devices, attached to the base support of the cameras (Figure 3B). In addition, in order to facilitate data recording and analysis, a transparent acrylic support was built on which the parameters and limits of angular inclinations of the body segments were printed (Figure 3B). The support that was fixed frontally to all cameras, with the exception of Camera 5 (overview), which was used only to support the researcher to reduce possible uncertainties during data collection.

A dental surface scaling time of 20 s was established for each dental surface, monitored by the researcher, and so each participant performed the dental scaling for a total time of 120 s. The outcome variable was defined as the sum of all “Awkward Postures” (yes/no) attributed to each of the 12 requirements assessed.

Table 1 presents the angular parameters applied to classify the postures of the head, neck, and trunk into three types according to international standards, as follows [26,29,30]:

- Neutral posture (acceptable)—when the inclinations were within the minimum limit;
- Moderate static posture (unacceptable)—when the maximum holding time (MHT) lasts for four or more seconds), and;
- Awkward static posture (unacceptable)—when the slopes are greater than the extreme limit.

MHT is a measurement related to static muscle work and represents the maximum time during which a static muscle load can be maintained while adopting moderate posture. ISO 11226 includes a relationship between inclination and maximum acceptable holding time, which was defined as 20% of maximum holding time—MHT [29,31,32]. Ohlendorf et al. considered that the static body posture is defined as a posture that is being held for more than four seconds [33].

Other studies considered the specificities and physiological requirements for the proper performance of tasks in dental practice, thus corroborating the application of these angular parameters and their relationships with the MHT and classification of the types of static postures [4,17,32,33,34,35].

Thus, in this study, to analyze head, neck, and trunk inclinations, the value of 20% of MHT was applied when the angular limits of neutral posture were exceeded, without, however, reaching the extreme angular limits of awkward postures.

Hence, in this study, four seconds was determined as MHT for moderate postures, a value that corresponds to 20% of the total dental scaling time for each dental surface.

To analyze the flexion and abduction of the upper arms, the relationship between inclination and maximum acceptable holding time specified by ISO 11226 was applied [29].

The parameters of back rounded backwards (C-shaped back), raised shoulders, flexion, and knee angle forearms and trunk-to-thigh angle were based on ESDE [17].

As can be seen in Figure 3G, dentists in both groups were provided with a dental stool (Manufacturer Dabi Atlante, standard model, with sloping seat front edge) that allows them to work with seat inclination between 90 degrees to 110 degrees in the popliteal region and between the thighs and abdomen. However, the training given to the intervention group emphasized the benefits of working with an angle of at least 110 degrees.

The analysis of the videos was performed by two researchers (ESO and AVSSC). To improve the reliability of the results, training and analysis calibration of ten (10) videos of simulated periodontal procedures were performed. The videos were recorded during data collection in phase 1. First, each of the 12 parameters were reviewed, discussed, and doubts about the classification criteria, such as neutral, moderate, and awkward posture were clarified, based on the parameters proposed by ESDE, ISO 11226, and DIN EN 1005-4. Next, by using a check-list of the 12 ergonomic parameters the 10 videos were evaluated separately by each of the researchers. In order to facilitate counting the maximum holding time (MHT) on each of the tooth surfaces, the video editing software AVS Video Editor V. 7.2.1 was used. Then, a meeting was held to present and discuss each classification attributed by the researchers to each of the 12 parameters evaluated in the 10 videos evaluated. The results presented by each researcher were compared and the differences were discussed until reaching a consensus, which was considered the “gold standard”. Cohen’s unweighted Kappa test was applied, obtaining values of 0.92 and 0.89 for the ESO and AVSSC researchers, respectively. These Kappa values revealed that the level of agreement reached was excellent and certified that the analysis criteria were properly applied in accordance with the criteria and parameters recommended by ESDE, ISO 11226, and DIN EN 1005-4. It should be clarified that one of the video evaluators (ESO) actively participated in the discussions and analyzes on the applicability of ISO 11226 to the conditions, specificities, and ways of working with dentists. This participation took place at the ESDE annual meetings held in 2002, 2003, 2005, and 2006 and provided in-depth knowledge about the adoption of a correct working posture, in accordance with ISO 11226 and DIN EN 1005-4. This allows us to infer that a larger number of evaluators would have a limited contribution to increase the reliability of the results.

### 2.6. Phase 2

This phase was carried out from September 2016 to February 2017.

IG ergonomic training (intervention) was carried out through theoretical activities, consisting of a presentation of the requirements for adopting a healthy work posture and presentation and provision of demonstrative video. The training activities were carried out in postgraduate clinics, in accordance with the European Society of Dental Ergonomics protocol [17].

Recording videos of the procedures performed by IG participants. Recording videos of the procedures were performed by the CG. To benefit all research participants, the CG (control) also received ergonomic training after the study was completed.

### 2.7. Data Analysis

IBM SPSS Statistics 27 was used for the exploratory and descriptive statistical analysis of the awkward posture total score and of the 12 requirements separately. The level of significance was set at α = 0.05 for all statistical tests performed. Since the awkward posture total score data were not normally distributed, the generalized linear models, by applying generalized estimated equation (GEE) with Poisson distribution and log link function, were used. The comparison graphs of the 12 requirements between groups, before and after training, were performed using mixed generalized linear models and settings between subjects were used as a random effect. The network analyses were conducted with RStudio (R Core Team, 2019), using R packages qgraph, huge, bootnet, and EGAnet. Estimating Ising models required supported methods to maximize pseudo-likelihood. Estimate type of network is Ising Networks.

## 3. Results

In the initial time, 720 observations were performed in the 2 groups, 525 (73%) out of presented awkward postures. In the final phase, 720 observations were also made, 403 (56%) out of showing awkward postures, of which 328 (72%) were of the control group and 75 (28%) of the intervention group. In total, comprising the two phases, 1440 observations were performed, with 528 (37%) from the intervention group and 912 (63%) from the control group.

Table 2 shows the statistical analysis of the effectiveness of the ergonomic training on the intervention group compared to that of the control group. The training was effective, showing a statistically significant difference (*p* < 0.001) when compared to the control group and intervention group before training.

**Figure 4:**
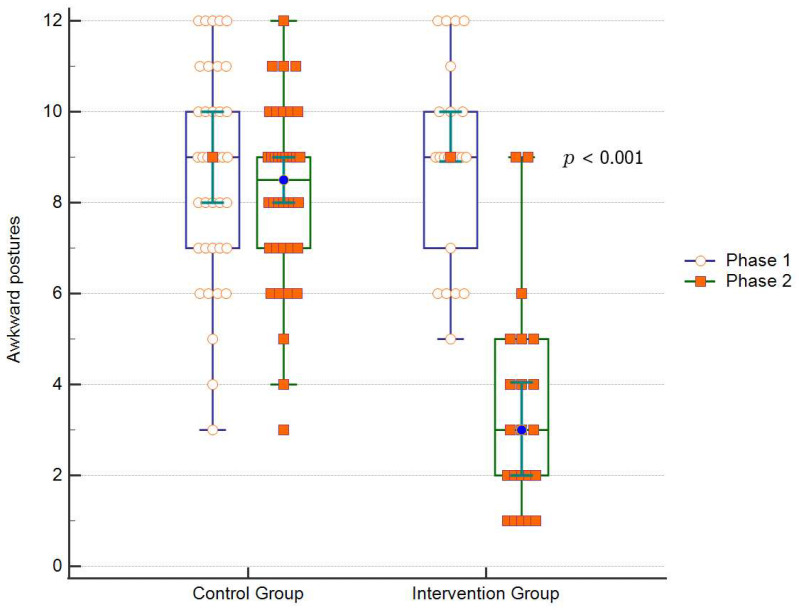
Distribution of the prevalence of awkward postures according to the groups, before (initial) and after (final) training.

The distribution of the prevalence of awkward postures according to the groups and also according to the initial and final phases is shown in Figure 4. Although there is a slightly larger amount in the initial intervention group, there were no significant differences among the groups initial control, initial intervention, and final control. On the other hand, there was a statistically significant difference after training of the intervention group, which resulted in a significant decrease in the adoption of awkward postures (Table 2 and Figure 4).

**Figure 5:**
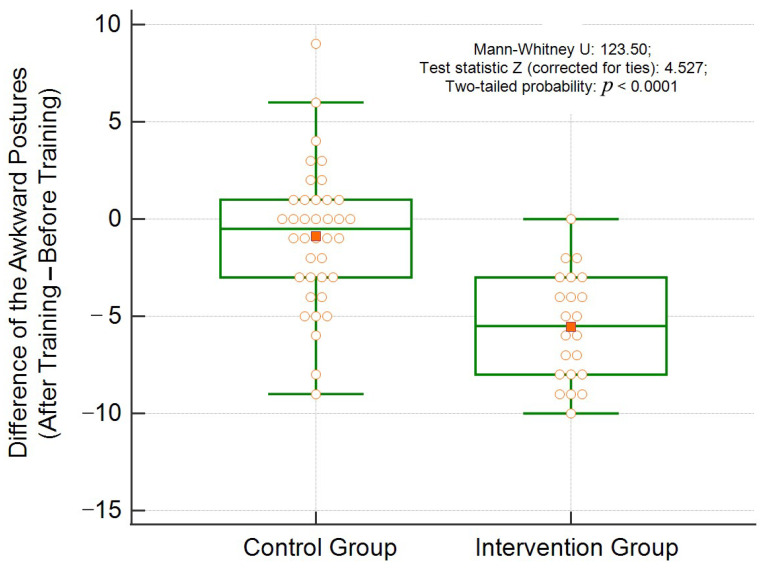
Distribution of the difference between the scores of awkward postures after and before training, according to the groups.

Figure 5 shows the distribution of the differences between the final minus initial score of the awkward postures according to the groups. The effectiveness of the training can be noticed since, in the intervention group, the differences were negatives (less than zero), and only one subject presented no improvement (difference equal to zero). On the other hand, in the control group, verified a substantial number of subjects with an equal or higher scores in the final phase was verified, resulting in a no or greater than zero difference.

**Figure 6:**
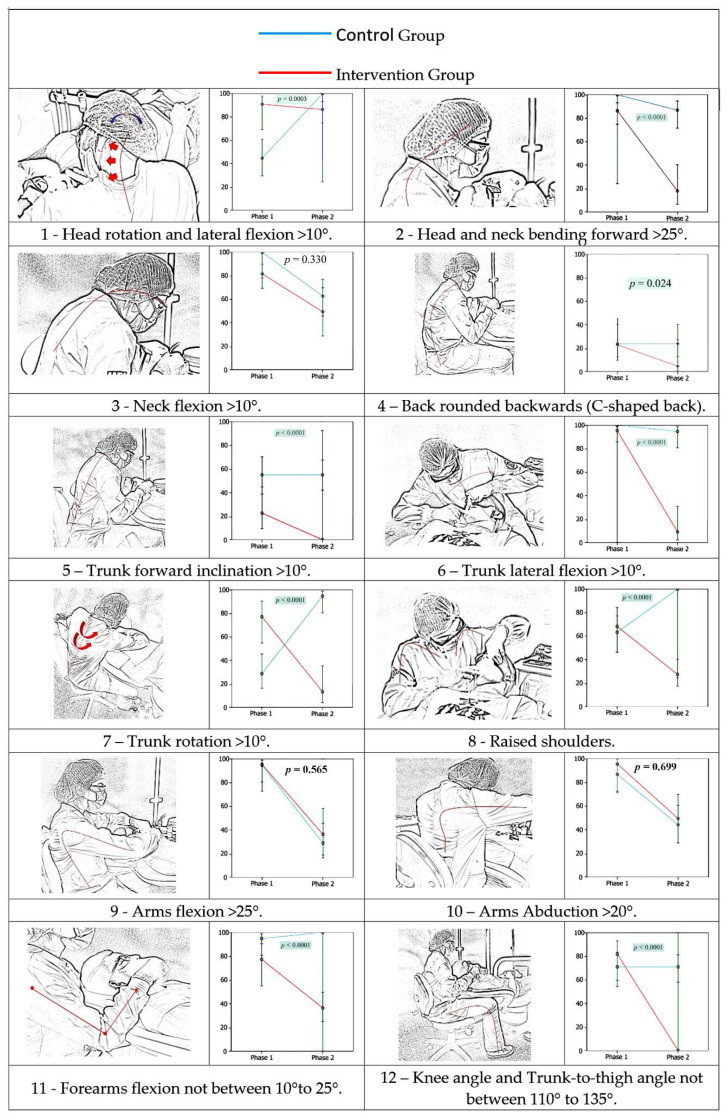
(**1**–**12**) Comparison of the awkward posture prevalence between groups and phases, according to 12 accessed parameters.

Images 1 to 12 of Figure 6 reveal that, in only three of the twelve posture requirements which evaluated the ergonomics training, there were no statistically significant differences between the control and intervention groups. Regarding inadequate neck flexion postures greater than 25 degrees (requirement 3), we found that there was a marked decrease in both the intervention and control groups, with no statistically significant difference (*p* = 0.330) after ergonomic training in the intervention group. A similar situation occurred with inadequate postures of upper arm flexion greater than 25 degrees (requirement 9) and upper arm abduction greater than 20 degrees (requirement 10), in which statistical significances were *p* = 0.565 and *p* = 0.699, respectively.

**Figure 7:**
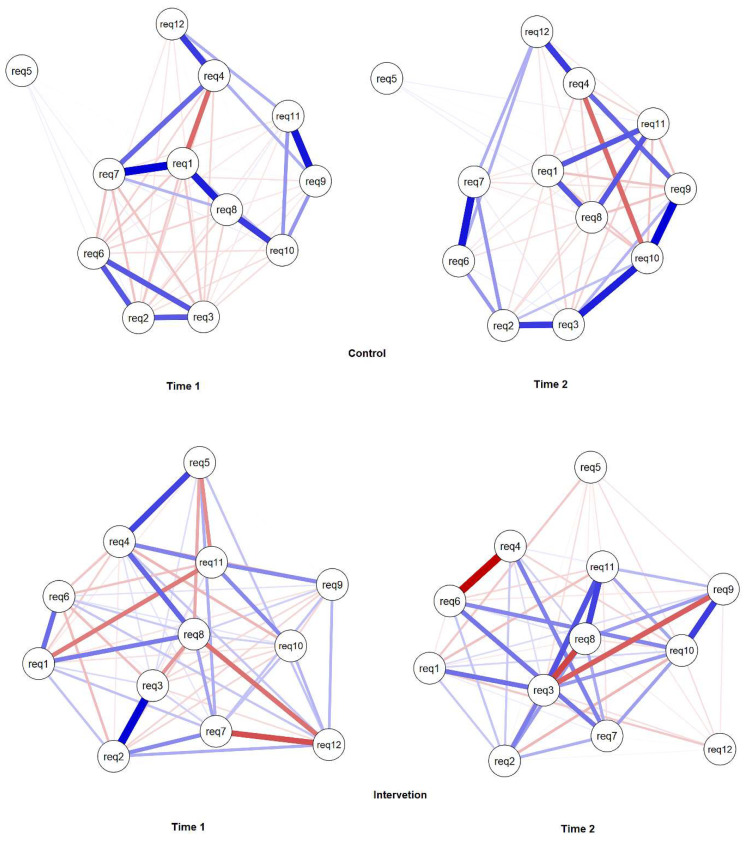
Estimated networks for ergonomics requirements according to body segments aspects, groups, and times.

**Figure 8:**
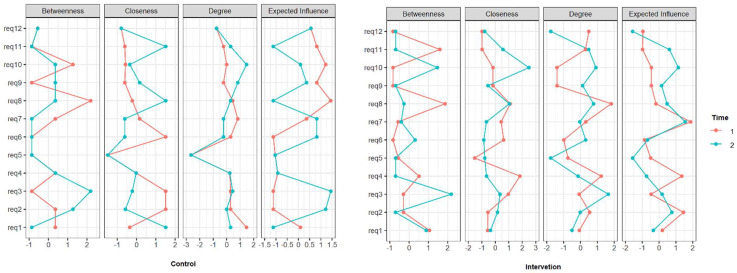
Networks analyses property.

Figure 7 and Figure 8 shows results from the analysis of networks, which were randomly distributed in the control and intervention groups at phase 1. Networks comprise graphical representations of the relationships (edges) between variables (nodes). Network analysis provides the capacity to estimate complex patterns of relationships and the network structure can be analysed to reveal core features of the network.

Neutral working posture is achieved through the balance of muscle forces that act in a kinematic chain. Hence, network analysis was applied to detect relationships between requirements, i.e., between types of awkward postures, according to phases and groups. Nodes represent variables (requirements) and undirected weighted blue edges show positive relationships (e.g., positive correlation/covariance between variables) while red ones show negative relationships. The strength of relationships is demonstrated by the thickness and colour density of the edge connecting the nodes; thicker denser coloured lines indicate stronger relationships.

Variable 8 (raised shoulders) had great importance in both groups, as seen in Figure 7. In relation to phase 1 and 2, in both groups, the relationships between the variables were maintained, only with a modicum of correlation forces, especially in the intervention group where the relationships were weaker.

## 4. Discussion

In the present study, the effectiveness of ergonomic training based on the parameters and requirements proposed by ESDE [17], ISO 11226 [29], and DIN EN 1005-4 [30] to decrease the prevalence of inadequate postures adopted by female dentists was verified, compared to those that apply the fundamentals of classical theory during the execution of scaling procedures periodontal. Findings of this study reveal that ergonomic training based on ESDE, ISO 11226, and DIN EN 1005-4 parameters and requirements resulted in a statistically significant overall decrease of 63 percent in the prevalence of awkward postures in relation to most requirements analysed.

The same did not happen with the control group, which, although it showed a small improvement in some requirements, was not statistically significant. This fact is important because, in the interval between the initial and final time, participants from both groups could have integrated new knowledge of ergonomics, which would be an external factor to the research. Thus, we can affirm that the difference found in the intervention group was not due to interference outside the study; otherwise, it would also have occurred in the control group.

In the initial phase, considering the two groups, there was a high frequency of non-recommended postures, corroborating the results found by several authors [13,18] who also found high percentages of non-recommended working postures practiced by professionals which are mainly responsible for high levels of WMSD prevalence [26]. Ergonomic training resulted in a decrease in the amount of non-recommended postures by 63% overall. There was a 100% reduction in the requirements for the frontal trunk flexion and angle in the popliteal region of less than 110 degrees and, therefore, a complete improvement due to the application of training.

According to Bridger et al. [37], this allows a neutral posture and concavity in the lower back of the spine to be maintained because it prevents the pelvis from tilting back more than 6.3 degrees. This result corroborates the results achieved by De Bruyne et al. [31,38], in which the influence of different types of owls on muscle activity and on lumbar posture was investigated. Twenty-five participants completed a sham dental procedure in a standard stool (90-degree popliteal region), a saddle-type stool, and the Ghopec stool (both allow more than 110 degrees in the popliteal region). The Ghopec stool, which meets the requirements proposed by ESDE, has a seat consisting of a horizontal rear part to support the pelvis and a downwardly sloping front part to support the upper legs, and a backrest for the legs. Vertically and horizontally adjustable back. The results of this study revealed that the lumbar posture was closer to neutral in the Ghopec stool, while sitting in a standard and in the saddle stools resulted in more flexed and extended lumbar postures, respectively.

Sitting at a 90-degree angle in the popliteal region (standard stool) resulted in greater activation of the back muscles while sitting at a 125-degree angle (saddle and Ghopec stool) activated the abdominal muscles more, although less in the presence of a backrest (Ghopec). According to de Bruyne et al., in order to maintain a neutral posture while performing dental procedures, the Ghopec owl is considered the most suitable design for the tasks performed [38]. The results of this study corroborate the findings of De Bruyne et al., (2016) and ESDE recommendations (2007) because they showed that trained dentists presented a significant decrease in the prevalence of awkward postures. This was mainly due to the change promoted by increasing the angle from 90 degrees to 110 degrees in the popliteal region and between the thighs and abdomen, with the dentist sitting on a dental stool provided with an inclination of the front of the seat by about 20 degrees and with an adequate backrest for the back.

The results found in relation to the requirements of flexion of the head and flexion of the neck showed an improvement of 56% and 68%, respectively. Lindegard et al. [18] concluded that the reduction in head and neck flexion can be achieved with the use of prismatic glasses, with the advantage that these devices do not have limitations related to focal length, as occurs with the use of magnifying glasses. Additionally, in the study by Hayes et al. [39], the researchers concluded that the use of magnifying glasses did not reach conclusive results about the benefits of using these devices in relation to improving head and neck posture.

The non-recommended positions of the head, trunk, shoulders, arms, and forearms could have achieved better results if the participants had correctly positioned the field of work. In this study, a mannequin was used, eliminating the professionals’ concern with the patient’s discomfort and, additionally, reducing interference regarding the possibilities of correct positioning of the work field, due to the inexistence of possible limitations inherent to the difficulties in moving the head of real patients. Despite this, similarly to what happens with a real patient, the research participants did not perform the proper movement of the mannequin’s head, which resulted in the incorrect positioning of the work field and the resulting adoption of an inadequate posture by the professional. In a study carried out with workers in a steel processing plant, Delleman [40] analysed the ideal mechanisms for adopting postures in relation to the work field. Guidelines on working height were formulated in order to minimize the load on the musculoskeletal system and it was found that the ideal distance from the working field to the visual field must be 35 cm. At this distance it is not necessary to flex the head and neck inappropriately to gain a view of the working field.

Botta et al. [41] conducted a cross-sectional study with dental students and found that they had a limited perception of risk factors. The lower the academic level of students, the greater their perception of risk factors that can contribute to musculoskeletal disorders. According to the authors, first-year students pay more attention to the risk factors they are exposed to when compared to senior students due to lack of clinical experience and concluded that more studies are needed to identify the most appropriate ergonomic program. In our study, we observed that dental surgeons, when receiving ergonomic training, obtain an improvement in relation to correct positioning, so training for the adoption of an ergonomic posture is an intervention strategy that needs to be carried out in order to reduce the prevalence of inappropriate postures.

This study was carried out with mostly female young professionals, who were students in specialization, Master’s, and doctoral courses in several areas. According to the Federal Council of Dentistry of Brazil (CFO), approximately 217,000 (60%) of the 361,981 registered dentists are women and the proportion of women has been increasing steadily in recent decades [42]. These data corroborate those reported by Ohlendorf et al. [26] which reveal the growing proportion of female dentists in several countries, and also demonstrate that women are more susceptible to developing MSD. Hence, in this study, the impact of training based on ISO 11226 and DIN EN 1005-4 parameters only on female dentists was chosen for study, aiming to reduce the confounding effect of a reduced number of male dentists registered in dental courses where the training was carried out. For this reason, the results found in this study are limited to female dentists, and a recommendation is to develop additional studies that also cover male dentists.

A high prevalence rate of awkward posture was found in both control and intervention groups, in the initial time, before training. This demonstrates that the knowledge of dental ergonomics based on the classical theory of the dentist’s sitting working posture acquired and retained in graduation was not sufficient for female dentists to adopt a neutral sitting working posture. It was also found that the performance of ergonomic training with emphasis on current knowledge can contribute to a decrease in the adoption of awkward postures. Most likely, Master’s and doctoral students will also be future professors and researchers; this fact reveals that, in order to change the current epidemiological profile of MSD in dental practitioners, the appropriate teaching of dental ergonomics needs to be stimulated and promoted in undergraduate, specialized, Master’s, and doctoral courses, thus corroborating the findings of Jianru et al. [14]. Additionally, the dental scaling procedures performed in this study are performed with high frequency in the practice of general dentistry and in the specialty of periodontics, which makes these findings of special importance for dental professionals in these specialties.

The analysis of the network offers the opportunity to advance in elucidating the dynamics of the muscular kinematic chain responsible for the posture adopted during the performance of dental treatments. Network analysis is a promising field of research should be applied in network analysis to longitudinal data [43,44]. Through this type of analysis, we seek to better understanding of the changes in the associations between the 12 posture requirements over time, that is, before and after training. In the present study, we observed the relationships between variables that had a slightly different initial distribution in the control and intervention group, but with strength in the variable and the correlation decreased from time 1 to 2 in the intervention group.

The results found through the network analysis revealed the existence of a significant relationship between the body segments evaluated and that the ergonomic training produced positive changes in this relationship. These findings corroborate Hokwerda et al. [21] who stated that, to perform dental procedures, it is necessary for the dentist to have adequate hand-eye coordination, achieved through a kinematic chain composed of several body segments that must move in an ideal coherence in the space before the upper body of the dentist. This kinematic chain consists of the fingers, hands, forearm and arm, upper body, shoulders, neck, and head, which incorporates the viewing direction of the eyes. In order to carry out the different tasks, visual, tactile, and proprioceptive perception is necessary. Furthermore, according to Hokwerda et al. [17], the information collected is processed and the output generated, directed towards the movement of the kinematic chain, with the hand and fingers at the end of the chain to carry out the tasks.

The network analysis in the IG revealed that working with raised shoulders (requirement 8) and neck flexion greater than 10 degrees (requirement 3) presented greater centrality, thus demonstrating a stronger level of relationship with the others posture requirements. After training, there was a significant inactivation of the negative relationship between requirements 8 and 12, revealing that the adoption of a posture with knee angle and trunk-to-thigh angle between 110° to 135° significantly contributed to reduce shoulder raising (Figure 6(12) and Figure 7). However, training did not significantly contribute to improvement in relation to neck flexion (requirement 3), which can be explained by its weak or non-existent relationship with requirement 12. This corroborates the recent findings of the study conducted by De Bruyne et al. [45] who demonstrated that just working seated higher is not enough to improve neck posture. In this study, dentists were not given the option to wear prismatic glasses or magnifying glasses. Hence, it was not possible to assess the corroboration with the findings of the study of the Lindegard et al. [18,46] which demonstrated that using glasses with a prism segment so that there is a reduction in the angle of the operator’s neck during the execution of dental treatment and also to reduce neck pain significantly more at follow up compared with the reference group.

This study highlighted the importance of using the network analysis method for a better understanding of the relationships of the body segments promoted by the kinematic muscle chain and its balance for the adoption of a neutral working posture. Additional studies using this method can contribute to deepen in the knowledge on the dynamics of the kinematic muscle chain when performing dental treatment.

Gathering and analysing data by using a control group in the initial and final phases allowed to minimize some limitations of possible external interference. It was not possible to perform blinding because our intervention required training for only one of the groups. The behaviour of the participants may have changed favourably, due to the awareness of participating in the research, as the participants were being observed and filmed, leading them to adopt a different posture from the usual one. This phenomenon is known as the Hawthorne effect, which may have produced a synergism effect, enhancing the decrease in the prevalence of non-recommended postures and, therefore, the results achieved may be overestimated [47]. However, some factors suggest that the chances that the results were influenced by factors outside the study are small. The fact that data collection and analysis was performed in both control and intervention groups, in the initial and final time, was a way to detect the impact of possible external interferences to the study, such as the access of research participants to classes, videos, and content about dental ergonomics. Additionally, it is also positive that the groups were quite homogeneous in relation to several factors such as gender (all participants were female), also due to similarity in terms of age, current context of life, daily routine divided between postgraduate studies, and providing care to their patients in the dental office.

Furthermore, the similar results obtained in the two groups in the initial time and also the similarity of the results in the control group in the initial and final times allow us to infer that it is unlikely that there was any kind of interference external to the research that interfered in only one of the groups. This was also due to the fact that both groups received guidance on the research objectives and on the conditions for developing the observations, and finally the fact that the participants of both groups were influenced by being aware of being filmed while performing dental scaling. Otherwise, there would have been a low prevalence of awkward postures also in the initial time.

This study was based on the assumption that the participants at the beginning of this study, both in the control group and in the intervention group, had not updated knowledge on the subject. Based on the so-called classical theory of sitting posture in dental work, research and teaching of dental ergonomics in dentistry courses in Brazil were very successful between the 70s and 90s. Research on dental ergonomics was accentuated. As a result, in most courses, the content taught today is practically the same as it was about 40 to 50 years ago, without therefore following the evolution of knowledge that took place in the last two decades, which was not effectively transferred to teaching practice by the great most teachers in the area in Brazil. The results achieved confirmed the premise that it is necessary to strengthen research and teaching of dental ergonomics for the dissemination of new knowledge in teaching practice and in the daily lives of future dentistry professionals. Therefore, in the current context of teaching dental ergonomics both in Brazil and internationally, applied training actually represents a paradigm shift between two opposing foundation theories, namely the classical theory and the one based on the new parameters based on ESDE, ISO 11226, and DIN EN 1005-4.

This study was conducted using a personalized methodology developed by its researchers due to the impossibility of accessing high technology, such as the use of accelerometers and advanced information technology systems. However, despite the simplicity of the method employed, all precautionary measures were taken to ensure the reliability of the angular and time measurements, which were confirmed by the excellent results achieved in the training and calibration process of the examiners. Thus, the use and presentation of a methodology with characteristics of simplicity, low cost, and the feasibility of obtaining high reliability results is a great contribution brought by this study. Such characteristics increase the possibilities that other researchers with similar conditions have the possibility of developing studies in the field of dental ergonomics, even if they have little or no access to resources and methods with high added technology and at high cost.

Another important contribution of this study, from the perspective of the researchers of this study, is the fact that its results reveal that practically all of the dentists are at high risk of developing MSD because they have neither access nor to current advances in ergonomic knowledge, nor to ergonomically improved equipment to minimize such risks. This study corroborates with studies published in the scope of ESDE and makes a significant contribution to the development of knowledge about dental ergonomics, considering the importance of knowing the real limitations in coping with and reducing the burden and suffering of dentists resulting from MSD.

Although the Nordic Musculoskeletal Symptoms Questionnaire was not applied, when questioned, all study participants declared that they had not had symptoms of WMSD, pain, or discomfort in the last twelve months. It is recommended that similar studies should be carried out using digital accelerometers, which allow for greater ease and increase objectivity regarding the measurement of inclinations in the various body segments. Thus, obtaining numerical variables in an automated way will allow the use of statistical techniques that have even detail and specificity regarding the benefits due to the training of dentistry professionals.

## 5. Conclusions

It was concluded that the ergonomic training based on the parameters of ISO 11226 and DIN EN 1005-4, as well as its adaptations to the dental practice provided by the European Society of Dental Ergonomics and other recent studies, contributed significantly to reducing the prevalence of awkward postures adopted by female dentists during the simulation of the basic periodontal procedures. However, it was not effective enough to improve the posture of the head and neck.

## Figures and Tables

**Table 1:** Parameters used as limits of inclination, according to the body segment and positioning of cameras used for evaluation [4,17,29,30,32,33,36].

Parameters According to Body Segment	Angle Range Values According to Ergonomic Standards			Camera Position
	Neutral	Moderate *	Awkward	
1—Head and neck rotation and/or lateral flexion	−10 to 10°	-	<−10° or >10°	Backward and/or Upper
2—Head and neck bending forward	0 to 25°	25 to 85°	<0° or >85°	Left
3—Neck flexion (sagittal)	0 to 25°.	25 to 45°	<0° or >45°	Left
4—Back rounded backwards (C-shaped back)	visual check of the back shape			Right
5—Trunk inclination (sagittal)	0 to 20° <0 full back support	20 to 60°	<0° or >60°	Right
6—Trunk lateral inclination	−10 to 10°	−10 to −20° & 10 to 20°	<−20° or >20°	Backward
7—Trunk rotation	−10 to 10°	-	<−10° or >10°	Upper
8—Raised shoulders	visual check of the shoulders shape			Backward
9—Upper arms flexion	0 to 20°	20 to 60°	>60°	Right
10—Upper arms abduction	0 to 20°	20 to 60°	>60°	Backward
11—Forearms flexion	10 to 25°	-	<10 or >25°	Right
12—Knee angle and trunk-to-thigh angle	110 to 135°	-	<110 or >135°	Right

*—Unacceptable if holding time ≥ 4 s.

**Table 2:** Comparation of estimated marginal means of non-compliance ergonomic postures, according to group and time.

Group	Time	Estimated Marginal Mean of Non-Compliance Score	*p*-Value *	Wald 95% CI for the Mean	
				Lower	Upper
Control	Initial	8.632	0.598	7.937	9.386
	Final	8.211		7.385	9.128
Intervention	Initial	8.955	<0.001	8.036	9.978
	Final	3.409		2.747	4.231

* Generalized linear models: generalized estimated equation. Poisson distribution with log link function.

## Data Availability

The data presented in the present study are available on request from the corresponding author.

## Supplementary Materials

The following are available online at https://www.mdpi.com/article/10.3390/ijerph182111217/s1, Table S1: CONSORT 2010 checklist of information to include when reporting a randomized trial.
